# Increased ROS levels activate AMPK-ULK1-mediated mitophagy to promote pseudorabies virus replication

**DOI:** 10.1186/s13567-025-01595-9

**Published:** 2025-07-21

**Authors:** Yuan Zhao, Xiaoyi Qi, Zhenbang Zhu, Wenqiang Wang, Wei Wen, Xiangdong Li

**Affiliations:** 1 Jiangsu Co-Innovation Center for Prevention and Control of Important Animal Infectious Diseases and Zoonoses, College of Veterinary Medicine, Yangzhou University, Yangzhou, China; 2 Joint International Research Laboratory of Agriculture and Agri-Product Safety, the Ministry of Education of China, Yangzhou University, Yangzhou, China; 3 Key Laboratory of Protection & Utilization of Biological Resources in Tarim Basin, College of Life Sciences, Tarim University, Alar, 843399 China

**Keywords:** Pseudorabies virus, reactive oxygen species, mitophagy, AMPK, mitochondria Ca^2+^, Nrf2

## Abstract

Increasing evidence has confirmed that oxidative stress plays a nonnegligible role in the viral pathogenic process. In this study, we investigated the role of reactive oxygen species (ROS) in the replication of pseudorabies virus (PRV). Our data showed that PRV infection initially enhanced the contact between the endoplasmic reticulum (ER) and mitochondria, leading to an upsurge of mitochondrial Ca^2+^ (mtCa^2+^) concentration, which resulted in the loss of mitochondrial membrane potential (MMP) and excessive ROS production. Instead of translocating it to the nucleus, PRV infection concurrently sequestered Nrf2 in cytoplasm impeding the efficient scavenging of intracellular ROS. The excessive ROS production and failure in ROS clearance contributed to the persistently high ROS levels during PRV infection. Furthermore, elevated ROS levels elicited activation of the AMPK-ULK1 axis, initiating PINK1-Parkin-dependent mitophagy that selectively degraded damaged mitochondria along with mitochondrial-localized mitochondrial antiviral signaling protein (MAVS). This process suppressed MAVS-mediated type I interferon responses by eliminating both dysfunctional mitochondria and their associated antiviral signaling platforms, thereby creating a cellular environment permissive to viral replication. Overall, our findings elucidated the mechanism by which ROS enables the virus to resist the host interferon immune response and provided a theoretical basis for ROS-based antiviral strategies.

## Introduction

Oxidative stress is a stress state that occurs when the balance of oxidation and anti-oxidation system breaks down. This is caused by an imbalance between the excessive production of cell—damaging free radicals, mainly reactive oxygen species (ROS), and the host's ability to neutralize them. For optimal living condition, viruses have developed tactics to trigger oxidative stress resulting in disrupting host antiviral mechanisms and hijacking the cellular processes for their proliferation. Meanwhile, ROS has been reported as vital contributor for viral proliferation and pathogenic processes. During Hepatitis B virus (HBV) infection, increased endogenous ROS levels have been shown to accelerate the pathogenic process of liver damage [1]. Influenza virus infection disrupts redox homeostasis, which makes the host epithelial cells more susceptible to viral infection and cell-to-cell transmission [2]. Herpesviruses, such as herpes simplex virus 1/2 (HSV-1/2), Epstein-Barr virus (EBV), pseudorabies virus (PRV), are neurotropic viruses that establish a lifelong latent infection in the nerve cells of the host and cause recurrent outbreaks. In a mouse model, recurrent HSV-1 infection induces oxidative modification of proteins and lipids in mouse brain, associated with the accumulation of the main Alzheimer’s disease pathognomic hallmarks and the development of neurodegeneration [3]. Clinical data showed high levels of oxidative stress in EBV-infected patients, and researchers have put forward a proposal that EBV ( +) tumors may be oxygen-driven tumors [4]. As a member of the *Herpesviridae* family, PRV is a highly contagious pathogen capable of infecting pigs and various wildlife species. Notably, since 2011, the emergence of mutant strains has led to increased virulence in pigs as classical vaccines failed to provide effective protection against them, resulting in damages to swine industry. PRV has been reported to exhibit a highly levels of oxidative stress both in vitro and vivo models [5, 6], and the alleviation of redox status by antioxidant impedes the viral replication [7, 8]. However, the mechanism whereby high levels of oxidative stress promote viral proliferation remains to be elucidated.

ROS are generated as by-products of various metabolic processes, particularly within the mitochondria during oxidative phosphorylation. Electrons derived from the oxidation of nutrients flow through the electron transport chain (ETC), which consists of multimeric protein complexes I–IV, to reduce oxygen and produce water. However, a few electrons will leak from the ETC and reduce molecular oxygen to superoxide. This superoxide can then be dismutated into hydrogen peroxide by the enzyme superoxide dismutase. H_2_O_2_ can further react to form hydroxyl radicals, and other ROS that participate in various cellular signaling processes and homeostasis [9]. While low levels of ROS are necessary for normal cellular signaling, excessive ROS can cause oxidative stress. Correspondingly, cells are equipped with an antioxidant defense system to counteract ROS, including enzymatic and non-enzymatic antioxidant systems as well as Nrf2 antioxidant system [10]. Given that the generation of reactive oxygen species has stimulative effects on viral replication, viruses have developed sophisticated strategies to trigger ROS production. Hepatitis C virus (HCV) infection triggers ROS production via the inhibition of electron transfer from respiratory complex I to the next electron carrier [11]. Human immunodeficiency virus (HIV) is reported to induce a high ROS level resulting from mitochondrial membrane potential (MMP) depolarization [12]. Additionally, HBV infection can induce ER stress, which causes calcium ions (Ca^2+^) from the ER to flow into mitochondria (mt), leading to mtCa^2+^ overload and thus excessive production of ROS [13]. For PRV infection, the underlying mechanism through which PRV infections give rise to elevated ROS levels remains unknown.

In this study, we demonstrate that PRV infection causes mtCa^2+^ overload and the inhibition of Nrf2-mediated antioxidant system, leading to the persistent existence of excessive ROS. Excessive ROS serves as an inducing factor to trigger mitochondrial damage and mitophagy for the self-replication. This study provides the explanation of oxidative stress mechanism caused by PRV, which is beneficial to understand the molecular mechanisms and dynamics of redox modulation and fully realize the therapeutic potential of antioxidants during viral infection.

## Materials and methods

### Chemical reagents

Reagents used were as follows: Reactive Oxygen Species Assay Kit (Beyotime, S0033S), N-acetyl-L-cysteine (NAC, Beyotime, S0077), Enhanced Mitochondrial Membrane Potential Assay Kit with JC-1 (Beyotime, C2003S), DAPI (Beyotime, C1002), Nuclear and Cytoplasmic Protein Extraction Kit (Beyotime, P0027), Cell Mitochondria Isolation Kit (Beyotime, C3601), CCK-8 Cell Counting Kit (CCK-8, Vazyme, A311-01), Dorsomorphin (MCE, HY-13418A), MCU-i4 (TargetMol, T9031), Rhod-2,AM (Yeasen, 40776ES50), Sulforaphane (MCE, HY-13755).

Antibodies used were as follows: GAPDH (CST, 5174), COX4 (CST, 4850), SQSTM1/P62 (CST, 8025), LC3B (CST, 3868), Parkin (CST, 4211), OPA1 (CST, 80471), DRP1 (CST, 8570), phospho-DRP1 Ser616 (CST, 4494), MFN1 (CST, 14739), Phospho-AMPKa (Thr172) (CST, 2535 T), Phospho-ULK1 (Ser555) (CST, 5869), β-actin (CST, 4970), MFN2 (Proteintech, 121861-AP), AMPK (Proteintech, 10929-2-AP), Tubulin (Proteintech, 11224-1-AP), Nrf2 (Proteintech, 805931-RR), Keap1 (Proteintech, 80744-1-RR), HO-1 (Proteintech, 66743–1-Ig), NQO1 (Proteintech, 67240-1-Ig), Phospho-PINK1 Ser228 (affinity, AF7081), Phospho-Parkin Ser65 (affinity, AF3500), TOM20 (Santa Cruz, sc-17764), ULK1 (Santa Cruz, sc-3909044), GCLC (santa, sc-390811).

### Cells and viruses

PK-15 cells were obtained from the American Type Culture Collection and cultured in Dulbecco’s modified Eagle medium (HyClone, SH30243.01) containing 10% fetal bovine serum (Lonsera, S711-001S) and 100 U/mL penicillin/0.1 mg/mL streptomycin at 37 °C with 5% CO_2_.

A recombinant virus rPRV HN1201-EGFP-Luc (G-PRV) was a generous gift from Prof. Beibei Chu at Henan Agricultural University [14], which was used in western blot and qRT-PCR. The wild-type PRV JS-2020 strain (W-PRV) was stored in our laboratory, which was isolated and identified from PRV-infected pigs in 2020 [15]. The W-PRV was used in immunofluorescence and flow cytometry.

### Mitochondrial membrane potential (MMP) assessment

Cells were incubated with 10 µM JC-1 probe for 20 min at 37 °C after virus infection or/and drugs treatment. Cells were collected to detect the fluorescence intensity by flow cytometer (Becton–Dickinson, LSRFortessa). The counts of MMP depolarization cells that exhibits lower red fluorescence and higher green fluorescence was calculated to indicate the degree of MMP depolarization. Three independent experiments were performed.

### ROS level and Mitochondrial calcium ions assessment

Cells were incubated with 10 µM DCFH-DA (ROS probe) or 1 µM Rhod-2, AM dye (mtCa^2+^ probe) for 20 min at 37 °C after virus infection or/and drugs treatment. Cells were collected to detect the fluorescence intensity by flow cytometer (Becton–Dickinson, LSRFortessa). The mean fluorescence intensity of cells was analyzed to indicate the changes of ROS and mitochondrial calcium ions. Three independent experiments were performed.

### Cell viability assessment

Cells were seeded in 96-well plates and treated with drugs. After indicated treatment, 10 µL CCK-8 Cell Counting Kit reagents were added to each well and incubated at 37 ℃ for 1.5 h. The absorbance was measured using microplate reader at 450 nm. Three independent experiments were performed.

### Small interfering RNA (siRNA) treatment

The siRNA for AMPK (Sequence 5′-3′ sense: GCUUGCCAAAGGAAUGAUUTT) was purchased from GenePharma (Shanghai, China). 20 nM of siRNAs were applied to transfect PK-15 cells with JetPRIME Transfection Reagent following the manufacturer’s specification. And then cells were infected with PRV to perform the indicated assays.

### Western blot

Cells were collected and lysed in RIPA buffer supplemented with 1 mM protease inhibitor PMSF to prepare total lysates. Mitochondrial lysates and nuclear lysates were prepared following the instructions of the commercial kit. After detecting the protein concentration using the bicinchoninic acid (BCA) method, protein samples were separated by SDS-PAGE gels and transferred to polyvinylidene fluoride (PVDF) membranes. Then, PVDF membranes were blocked with 5% skim milk for 90 min at room temperature (RT), incubated with adequate primary and secondary antibodies, and finally developed with chemiluminescent substrates. The protein bands were detected on Tanon 5200 Multi (Shanghai, China) using ECL Kit and quantified by Image J analysis. The density of each band was normalized to its respective loading control (β-actin or GAPDH or tubulin).

### RNA extraction and quantitative real-time PCR (qRT-PCR)

Total RNA was extracted with TRIzol reagent (Tiangen, DP424) and subjected to reverse transcription with HiScript III RT SuperMix for qPCR (Vazyme, R323-01). PCR reactions were performed with ChamQ Universal SYBR qPCR Master Mix (Vazyme, Q711) using ABI QuantStudioTM 3 (Applied Biosystems, CA, USA). Relative expressions of specific genes were calculated by 2^−ΔΔCt^ method by normalizing to the house-keeping gene (β-actin) expression. Primers were designed as follows and synthesized by Sangon Biotech (Shanghai, Co, Ltd.). *NFE2L2* (F: tccttcagcagcatcctctc; R: cacggtggtcttggttgaag), *HMOX1* (F: aagattgctcagaaggccct; R: ttagtgtcctgggtcagcag), *NQO1* (F: tggccgaacaaaagaaggtg; R: gcagagagtacatggagcca), *GCLC* (F: aggcatcgatcatctcctgg; R: gagtttggaggaggaggctt).

### Immunofluorescence staining

Cells were treated with indicated design, then fixed in 4% paraformaldehyde for 8 min, permeabilized with 0.1% Triton X-100 in PBS for 15 min, blocked with 2% bovine serum albumin in PBS for 1 h at room temperature, and incubated with primary antibodies at 4 °C overnight. After washing with PBS, cells were incubated with secondary body at RT for 1 h, followed by staining with DAPI. Finally, the slides were observed under a confocal microscope (ZEISS, LSM 880NLO) with a 63 × or 100 × oil immersion objective. The images were analyzed using ImageJ software.

### Viral titer assays

Cells were seeded in 96-well plates and treated with serial diluted samples. After 72 h of incubation, the Reed and Muench method was used to calculate the 50% tissue culture infective dose titer (TCID_50_).

### Statistical analysis

The results are expressed as the mean ± SD values. Significant differences among groups were determined with one-way analysis of variance followed by a Tukey test using statistical software GraphPad Prism. Values of *P < 0.05, **P < 0.01, and ***P < 0.001 were considered significant, and ns indicates a lack of significant difference.

## Results

### Increased ROS levels benefit PRV replication

**Figure 1:**
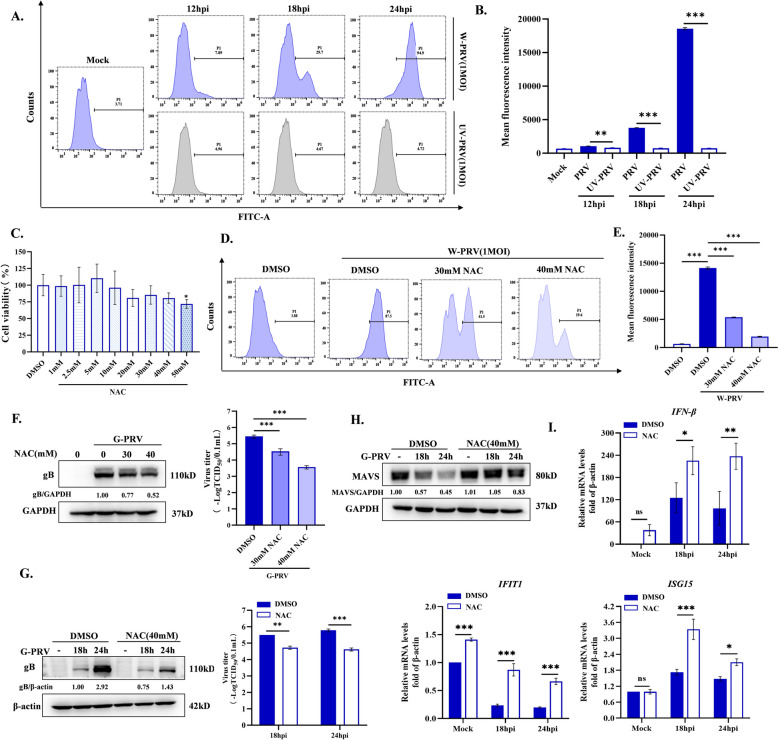
**Effects of ROS levels on PRV replication. A, B** Cells were infected with W-PRV or treatment with UV-PRV (equivalent of MOI = 1) at 12, 18, and 24 hpi. ROS levels were detected by flow cytometry using DCFH-DA probe and mean fluorescence intensity was measured. **C** Cells were treated with different concentrations of NAC for 24 h. Cell viability was detected by CCK-8. **D, E** ROS levels were detected by flow cytometry using DCFH-DA probe upon infection with 1MOI W-PRV or/and treated with 30 mM and 40 mM NAC for 24 h. Mean fluorescence intensity was measured. **F** Cells were infected with 1MOI G-PRV or/and treated with 30 mM and 40 mM NAC for 24 h. Viral gB protein level was detected by western blot and viral titers in cellular supernatants were detected by TCID_50_ assay. Cells were infected with 1MOI G-PRV or/and treated with 40 mM NAC for 18 h and 24 h. **G** Viral gB protein level was detected by western blot and viral titers in cellular supernatants were detected by TCID_50_ assay. **H** MAVS protein level was detected by western blot. **I** *IFN-β*, *IFIT1*, *ISG15* mRNA levels were detected by qRT-PCR. *, **, and *** indicate statistically significant differences with *P* < 0.05, *P* < 0.01, and *P* < 0.001, respectively.

Previous studies reported that increased ROS levels are beneficial to viral replication during infection [16]. PRV induced a large generation of ROS after 12 h of infection, but UV-inactivated virus did not (Figure 1A and B), suggesting that PRV infectious process is essential for the production of ROS. To investigate the role of ROS on the viral replication, we utilized N-acetyl-L-cysteine (NAC) to eliminate PRV-triggered ROS. Within the concentration ranges where cell survival was not affected, NAC treatment efficiently removed PRV-triggered ROS production (Figure 1C and E) and significantly decreased viral gB protein expression and virus titers in supernatants in a dose- and time-dependent manner (Figure 1F and G). Our previous study showed that PRV infection causes mitophagic degradation of MAVS and inhibits MAVS-mediated IFN-I responses, which contributes to viral replication [17]. Therefore, we hypothesized that NAC treatment-mediated inhibition of viral replication might be related to the restoration of the interferon response mediated by MAVS. As shown in Figure 1H and I, NAC treatment restored the MAVS protein level and increased the expression of its downstream IFN-I genes (IFN-β, the IFN-stimulated genes tetratricopeptide repeats 1, IFIT1 and ISG15) compared to the group with only PRV infection. These results indicate that the promotion of increased ROS levels by PRV initiates the mitophagic degradation of MAVS.

### Increased ROS level triggers mitochondrial damage and mitophagy during PRV infection

**Figure 2:**
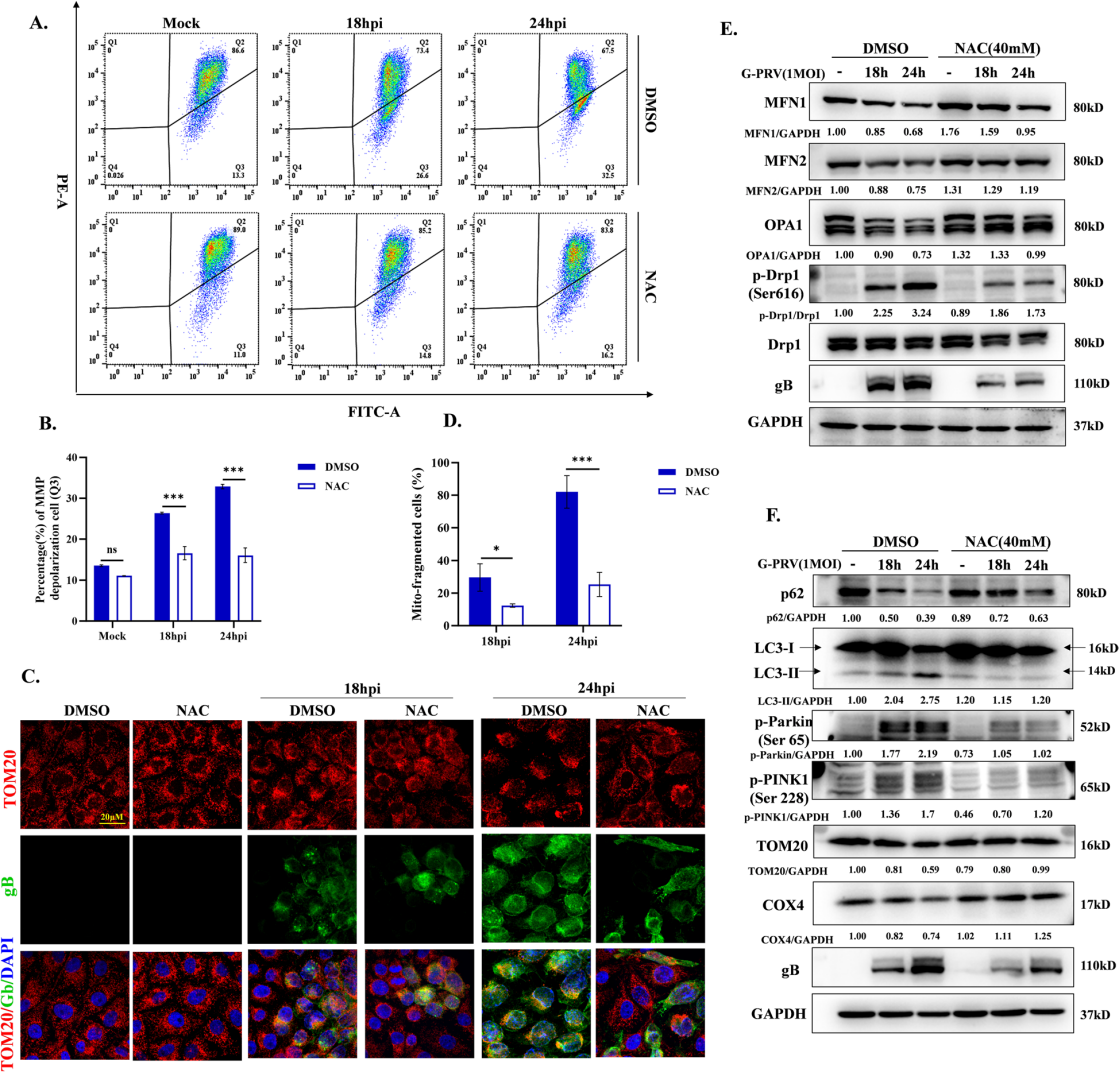
**Effects of NAC on mitochondrial membrane potential and morphology.** Cells were infected with 1MOI W-PRV or/and treated with 40 mM NAC for 18 h and 24 h. **A**–**B** MMP was detected by flow cytometry using the JC-1 probe. The counts of cells whose MMP (Q3 area) was depolarized were quantitated. **C, D** Mitochondrial network morphology (TOM20 labeled, red) was observed by confocal microscopy. The proportion of cells whose mitochondria manifested fragments were quantitated. Cells were infected with 1MOI G-PRV or/and treated with 40 mM NAC for 18 h and 24 h. **E** MFN1, MFN2, OPA1, Drp1, p-Drp1(s616) and gB protein levels were detected by western blot. **F** p-PINK1(Ser228), p-Parkin (Ser65), TOM20, COX4, p62, LC3 and gB protein levels were detected by western blot. * and *** indicate statistically significant differences with *P* < 0.05 and *P* < 0.001, respectively. ns indicates not statistically significant.

Excessive ROS has been recognized as the inducing factor that induces mitochondrial damage and mitophagy. To confirm whether mitophagy induced by PRV infection is associated with increased ROS levels, 40 mM NAC was utilized to carry out following experiment. Flow cytometry results showed that NAC treatment significantly improved PRV-reduced MMP (Figure 2A and B). Fluorescence microscopy images showed that NAC treatment alleviated the damage of mitochondrial morphology by PRV (Figure 2C and D). Mitochondrial morphology is regulated by the balance of the mitochondrial dynamic, i.e., fission and fusion of mitochondria. Western blot showed that NAC treatment increased fusion protein MFN1, MFN2 and OPA1 expression and decreased fission protein Drp1 phosphorylation at serine 616 during PRV infection, suggesting that inhibition of ROS production rescued PRV-disturbed unbalance of mitochondrial fusion and fission (Figure 2E). Finally, we detected the effects of NAC on PRV-induced PINK1-Parkin-mediated mitophagy [17]. As shown in Figure 2F, NAC treatment inhibited PRV-induced PINK1 and Parkin phosphorylation and reversed p62, LC3-II, COX4 and TOM20 protein expression. Taken together, these results demonstrate that increased ROS level aggravates mitochondria damage resulting in mitophagy occurrence.

### Increased ROS levels activate mitophagy via AMPK-ULK1 signaling pathway to promote PRV replication

**Figure 3:**
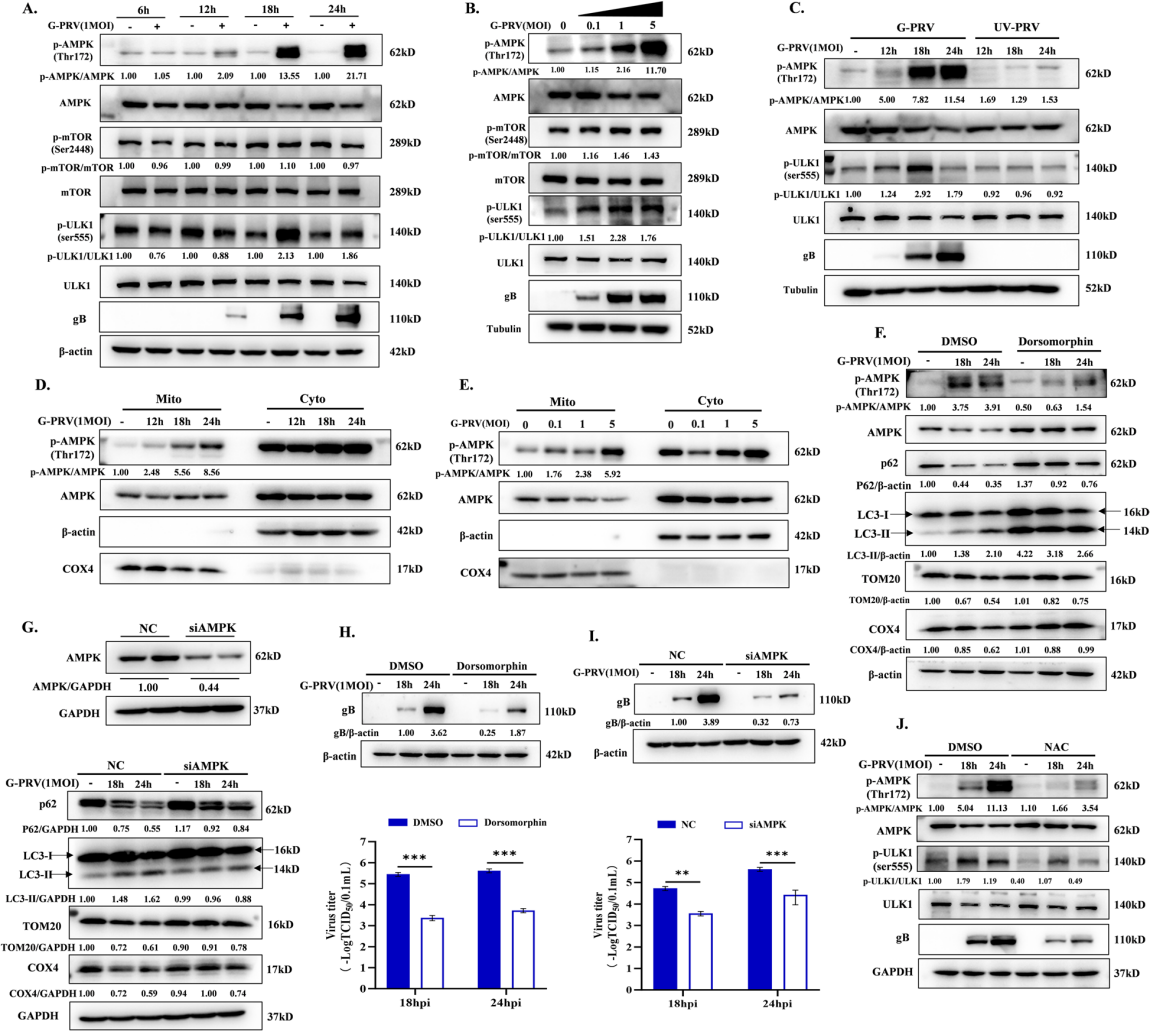
**Role of AMPK-ULK1 signaling pathway on PRV-induced mitophagy and viral replication. A-B** Cells were infected with 1MOI G-PRV for 6, 12, 18, and 24 hpi **(left)** or 0.1, 1, 5 MOI G-PRV for 18 h **(right).** AMPK, p-AMPK(Thr172), mTOR, p-mTOR (Ser2448), p-ULK1(Ser 555), ULK1 and gB protein levels were detected by western blot **(C)** Cells were infected with G-PRV or treated with UV-PRV (equivalent of MOI = 1) at 12, 18, and 24 hpi. AMPK, p-AMPK(Thr172), p-ULK1(Ser 555), ULK1 and gB protein levels were detected by western blot. **D-E** p-AMPK (Thr 172) and AMPK protein levels in mitochondrial and cytoplasmic lysates were analyzed by western blot upon G-PRV infection (MOI = 1) at 12, 18, and 24 hpi or 0.1, 1, 5 MOI G-PRV for 18 h. Cells were infected with 1MOI G-PRV or/and treated with dorsomorphin (30 µM) for 18 and 24 h. **F** AMPK, p-AMPK(Thr172), p62, LC3-II protein levels, TOM20 and COX4 levels were detected by western blot. **H** Viral gB protein level was detected by western blot. Viral titers in cellular supernatants were detected by TCID_50_ assay. Cells were transfected siRNA (negative control, NC and siAMPK) for 24 h and then infected with 1 MOI G-PRV for 18 h and 24 h. **G** Knockdown efficiency of AMPK was determined by western blot **(upper).** p62, LC3-II, TOM20 and COX4 protein levels were detected by western blot **(below)**. **I** The gB protein levels and viral titers in cellular supernatants were determined by western blot and TCID_50_, respectively. Cells were infected with 1MOI G-PRV or/and treated with 40 mM NAC for 18 h and 24 h. **J** AMPK, p-AMPK(Thr172), p-ULK1(Ser 555), ULK1 and gB protein levels were detected by western blot. **, and *** indicate statistically significant differences with *P* < 0.01, and *P* < 0.001, respectively.

ROS have been reported to activate AMP-activated protein kinase (AMPK) to regulate mitophagy and AMPK plays an important role in the maintenance of mitochondrial homeostasis [18]. During MMP depolarization, AMPK is activated by the phosphorylation at Thr172 and then ULK1 is phosphorylated at Ser555 by AMPK to promote mitochondrial fission and PINK1-Parkin-mediated mitophagy [19, 20]. Given the close connection between AMPK, mitochondrial function, and ROS, we further carried out experiments aiming to elucidate the causal relationship between ROS and AMPK in PRV-induced mitophagy. As shown in Figure 3A and B, PRV infection significantly increased the phosphorylation of AMPK at Thr172 and ULK1 at Ser 555 and there was no effect on mTOR activity. We also confirmed that the viral replication was a prerequisite for AMPK activation compared to the group with PRV infection (Figure 3C). Meanwhile, the activity of mitochondria-localized AMPK (mitoAMPK) has been confirmed as an important inducing factor for mitochondrial fission and mitophagy [21]. The AMPK activity in mitochondrial lysates was also detected during PRV infection. As shown in Figure 3D and E, the phosphorylation of mitoAMPK at Thr172 was increased in a dose- and time-dependent manner. To determine the role of AMPK on PRV-induced mitophagy, we utilized AMPK inhibitor dorsomorphin and AMPK knockdown (siAMPK) to inhibit AMPK activity. As shown in Figure 3F and G, 30 µM dorsomorphin treatment and AMPK knockdown blocked PRV-induced autophagy process and mitochondria degradation. Meanwhile, both inhibition strategies decreased gB protein level and viral titers in supernatant (Figure 3H and I). Finally, we found that NAC treatment inhibited PRV-induced AMPK and ULK1 phosphorylation (Figure 3J). Collectively, these results demonstrate that ROS acts as an inducing factor to activate AMPK-ULK1 signaling and then subsequently leads to PINK1-Parkin-mediated mitophagy.

### PRV-increased ROS levels result from mtCa^2+^ overload

**Figure 4:**
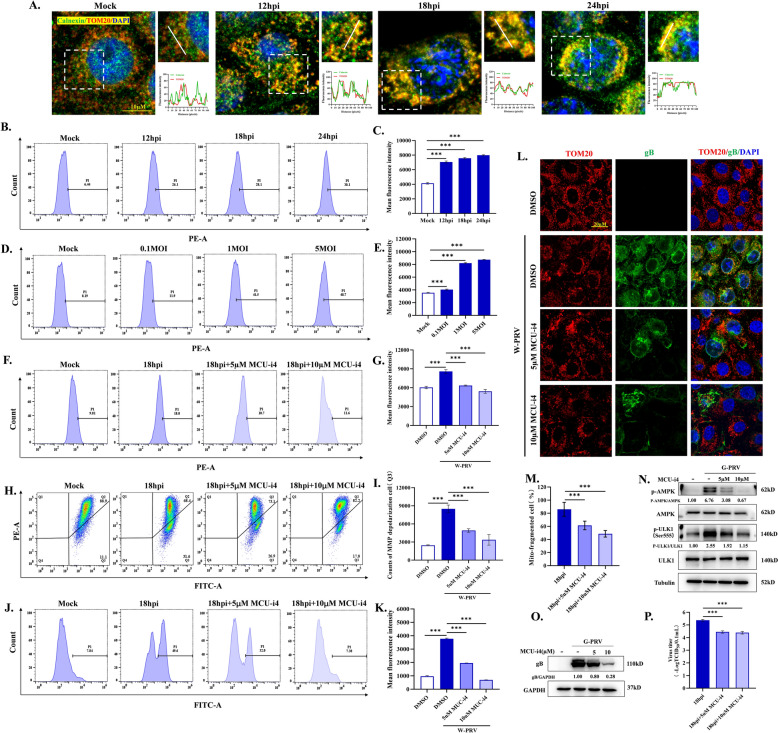
**Role of mitochondrial calcium on PRV-induced increased ROS levels and mitochondria damage. A** Colocalization of mitochondria (TOM20, red) with ER (calnexin, green) was visualized by confocal microscopy. **B-E** Cells were infected with 1MOI W-PRV for 12, 18, and 24 hpi **(upper)** or 0.1, 1, 5 MOI G-PRV for 12 h **(below).** mtCa^2+^ was detected by flow cytometry using Rhod-2AM probe and mean fluorescence intensity was measured. Cells were infected with 1MOI W-PRV or/and treated with 5 µM or 10 µM MCU-i4 for 18 h. **F-G** mtCa^2+^ levels were detected by flow cytometry using Rhod-2AM probe and mean fluorescence intensity was measured. **H-I** MMP was detected by flow cytometry using the JC-1 probe. The counts of cells whose MMP (Q3 area) was depolarized were quantitated. **(J-K)** ROS levels were detected by flow cytometry using DCFH-DA probe and mean fluorescence intensity was measured. **L-M** Mitochondrial network morphology (TOM20 labeled, red) was observed by confocal microscopy. The proportion of cells whose mitochondria manifested fragments were quantitated. **N** AMPK, p-AMPK(Thr172), p-ULK1(ser 555) and ULK1 protein levels were detected by western blot. **O-P** The gB protein levels and viral titers in cellular supernatants were determined by western blot and TCID_50_, respectively. *** indicate statistically significant differences with *P* < 0.001, respectively.

Since ROS has a significant promotion effect on viral replication, what specific mechanism does PRV use to cause ROS levels to increase? mtCa^2+^ dysregulation has been closely associated with abnormal ROS production. Under stressful conditions, mitochondria will excessively uptake Ca^2+^ from the ER, which is the main calcium storage organelle and whose function can influence the concentration of Ca^2+^ in the mitochondria, finally resulting in ROS production and mitochondrial damage. As shown in Figure 4A, PRV infection enhanced the contact between ER and mitochondria by the evidence of co-localization of the ER and mitochondria. We utilized Rhod-2AM probe to detect the changes of mtCa^2+^. Flow cytometry results showed that PRV infection caused mtCa^2+^ overload by the evidence of increase of the concentration mtCa^2+^ in a time- and dose-dependent manner (Figure 4B-E). The mtCa^2+^ uptake of calcium is dependent on mitochondrial calcium uniporter (MCU) [22]. To verify whether mtCa^2+^ overload is responsible for the subsequent increase in ROS and following events, we used MCU-i4, an MCU inhibitor, to inhibit mtCa^2+^ uptake. PRV-increased mtCa^2+^ was obviously reduced by MCU-i4 (Figure 4F and G). As shown in Figure 4H-K, MCU-i4 alleviated the depolarization of MMP and reduced the production of ROS compared to PRV group. The damage of mitochondrial morphology was mitigated by MCU-i4 compared to PRV group (Figure 4L and M). Further, we detected the effects of MCU-i4 on AMPK-ULK1 signaling pathway and the viral replication. The inhibition of mtCa^2+^ uptake suppressed PRV-induced AMPK and ULK1 phosphorylation and decreased the gB protein level and virus titers (Figure 4N and P). Given that, mtCa^2+^ overload serves as an inducing factor to damage mitochondria and stimulate mitophagy occurrence.

### The imbalance of Nrf2-driven antioxidant response is contributing to ROS accumulation during PRV infection

**Figure 5:**
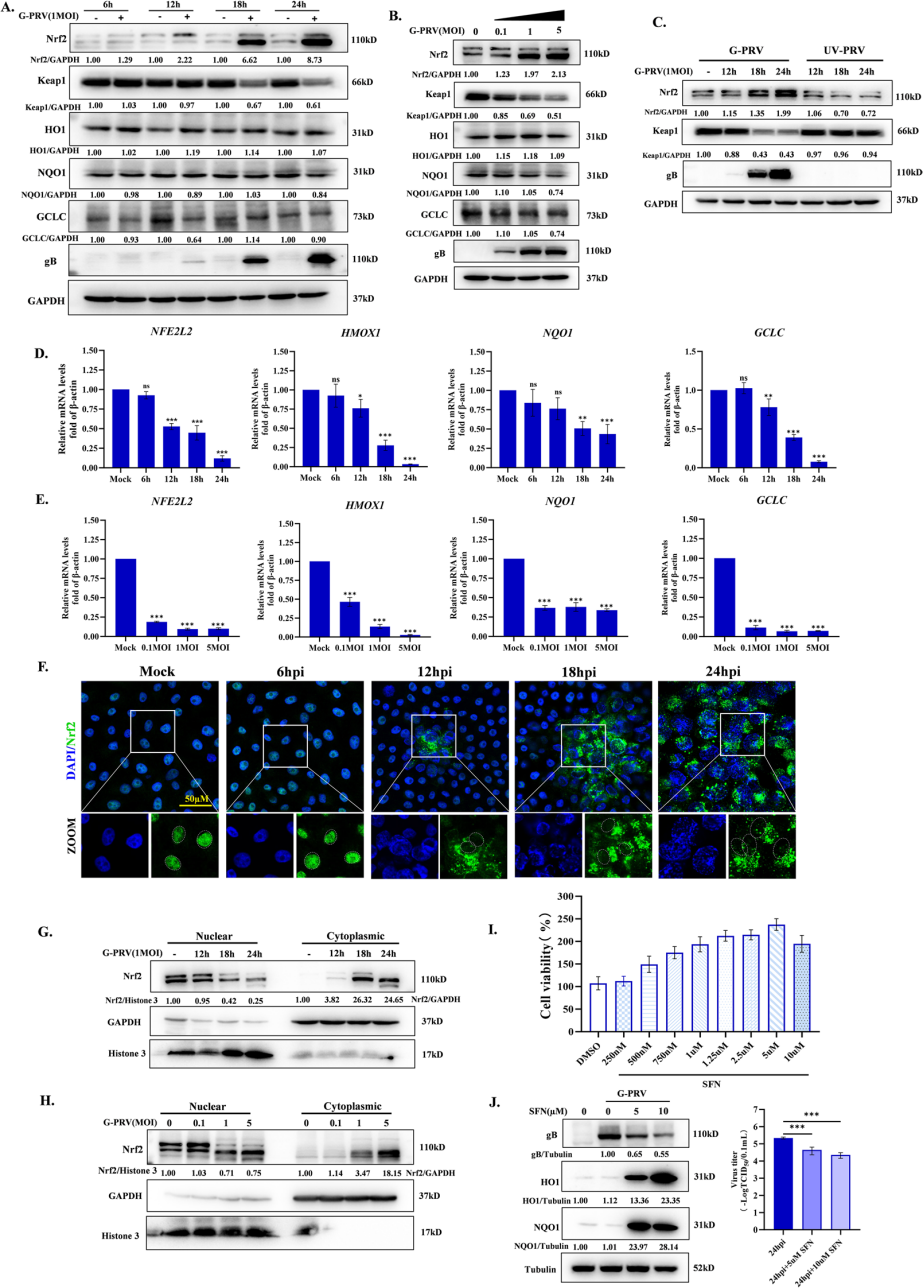
**PRV infection inhibited the production of Nrf2-mediated antioxidant enzyme. A-B** Cells were infected with 1MOI G-PRV for 6, 12, 18 and 24 hpi** (left)** or 0.1, 1, 5 MOI G-PRV for 24 h **(right).** Nrf2, Keap1, HO1, NQO1 and GCLC protein levels were detected by western blot. **C** Cells were infected with G-PRV or treatment with UV-PRV (equivalent of MOI = 1) at 12, 18, and 24 hpi. Nrf2 and Keap1 protein levels were detected by western blot. **D-E** Cells were infected with 1MOI G-PRV for 6, 12, 18, and 24 hpi **(upper)** or 0.1, 1, 5 MOI G-PRV for 24 h **(below)**. *NFE2L2*, *HMOX1*, *NQO1* and *GCLC* mRNA levels were detected by qRT-PCR. **F** The subcellar localization of Nrf2 was detected by immunofluorescence upon G-PRV infection (MOI = 1) at 6, 12, 18, and 24 hpi. **G-H** Nrf2 protein levels in nuclear and cytoplasmic lysates were analyzed by western blot upon G-PRV infection (MOI = 1) at 12, 18, and 24 hpi **(upper)** or 0.1, 1, 5 MOI G-PRV for 24 h **(below). I** Cells were treated with different concentrations of SFN for 24 h. Cell viability were detected by CCK-8. **J** Cells were infected with 1MOI G-PRV or/and treated with 5 µM or 10 µM SFN for 24 h. HO1, NQO1 and gB protein levels were detected by western blot. Viral titers in cellular supernatants were determined by TCID_50_. *, **, and *** indicate statistically significant differences with *P* < 0.05, *P* < 0.01, and *P* < 0.001, respectively. ns indicates not statistically significant.

In response to increased ROS levels, cells naturally initiate antioxidant system to antagonize oxidative damage. Nrf2 acts as a key regulator of the cellular redox-responsive system that plays a protective role for cells against various virus-caused oxidative damages [23]. Under physiological conditions, inactive Nrf2 mainly binds to its inhibitor Keap1 in the cytoplasm and is rapidly degraded by the ubiquitin proteasome pathway. When ROS is increased, activated Nrf2 uncouples from Keap1 and translocates to the nucleus combined with Maf protein to form heterodimer. NRF2-MAF complexes bind to antioxidant response elements (AREs) to drive the transcription of phase II metabolic enzymes (NAD(P)H:quinone oxidoreductase 1, NQO1), antioxidant enzymes genes(heme oxygenase 1, HO1; glutamate-cysteine ligase catalytic subunit, GCLC) to mitigate and recover from oxidative damage [24]. As shown in Figure 5A-C, PRV, rather than UV-PRV, infection increased Nrf2 protein level and decreased Keap1 protein level in a time- and dose-dependent manner. It seemingly suggested PRV infection promoted Nrf2 expression and made Nrf2 uncouples with Keap1 and translocation to the nucleus. However, there was no increased in the downstream effect protein of Nrf2, HO1, NQO1 and GCLC protein levels. qRT-PCR results showed that PRV infection inhibited *NFE2L2*, *HMOX1*, *NQO1* and *GCLC* mRNA levels in a time- and dose-dependent manner (Figure 5D and E). Further, western blot and IFA results showed that PRV infection reduced Nrf2 translocation in time- and dose-dependent manner, which suggested that PRV infection didn’t activate Nrf2 to bind AREs to drive the transcription of antioxidative genes (Figure 5F–H). Sulforaphane (SFN), a powerful activator of Nrf2 signaling pathway, can interact with specific cysteine residues of Keap1 to change the conformation of Keap1, resulting in the dissociation of Nrf2 from the Nrf2-Keap1 complex and promoting the translocation of Nrf2 to the nucleus [25]. As shown in Figure 5I and J, within the concentration ranges where cell survival was not affected, 5 µM and 10 µM SFN obviously increased HO1 and NQO1 expression during PRV infection, indicating the activation of Nrf2 signaling pathway by SFN. Meanwhile, SFN also inhibited viral gB protein and viral titers in supernatant. It suggested that Nrf2 antioxidant system played an important role in antagonizing the process of antiviral infection. Therefore, we concluded that PRV infection inhibits Nrf2 translocation into the nucleus and the expression of downstream antioxidative genes, resulting in consistent ROS accumulation.

## Discussion

**Figure 6:**
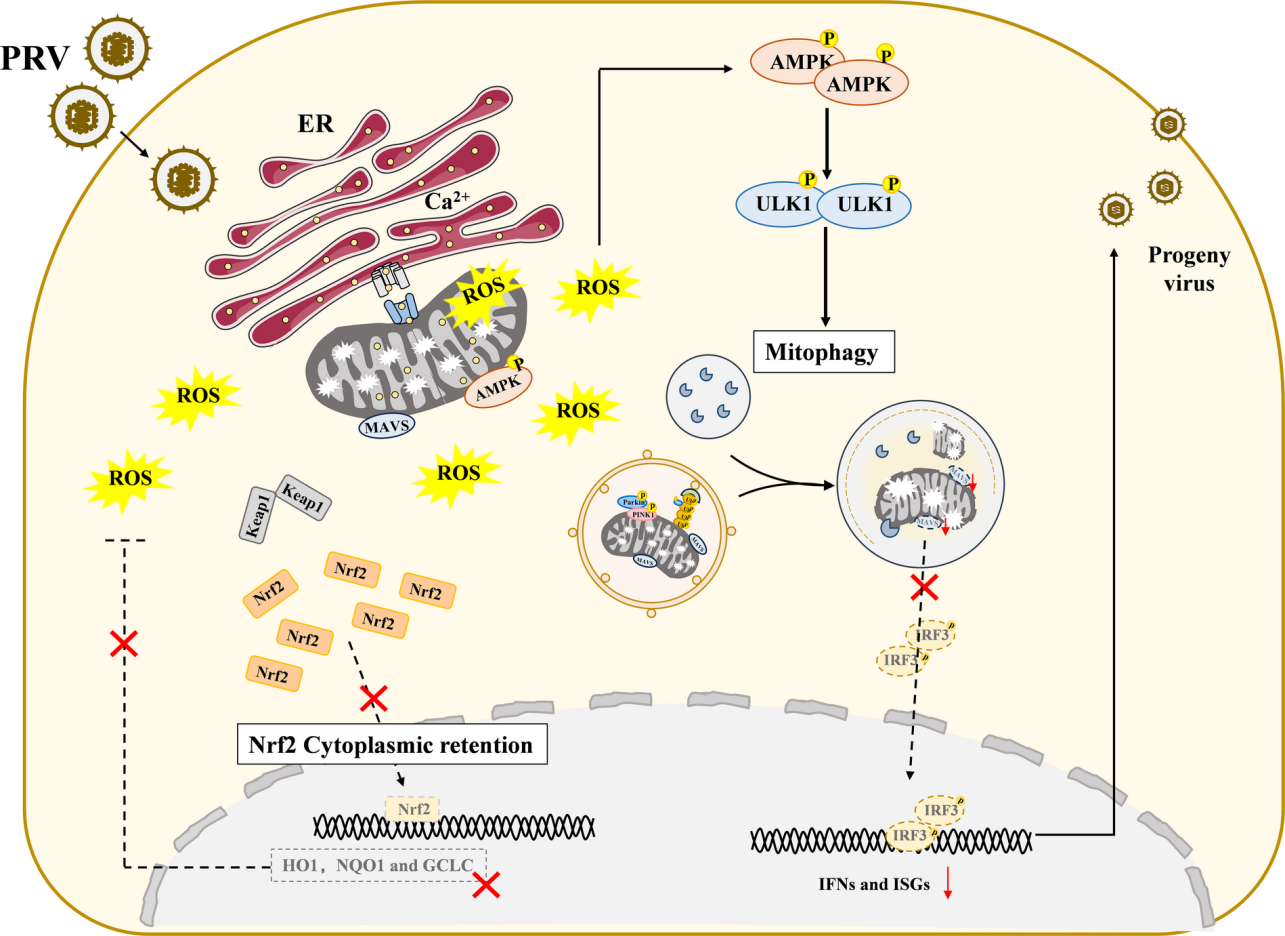
**Graphical diagram of the role of PRV-increased ROS levels on mitochondria damage, mitophagy and viral replication.** PRV infection enhances contact between the ER and mitochondria, leading to mitochondrial calcium overload and ROS production. Additionally, PRV infection sequesters the antioxidative transcription factor Nrf2 in the cytoplasm, impairing ROS clearance and promoting intracellular accumulation. Elevated ROS levels disrupt mitochondrial morphology and function, activating the AMPK-ULK1 axis and triggering PINK1-Parkin-dependent mitophagy. This process degrades damaged mitochondria and MAVS, thereby creating a cellular environment conducive to viral replication.

Virus infection can create an optimal environment for its own proliferation with the remodeling of intracellular microenvironment. ROS is a key regulator of cellular redox signaling, and its homeostasis plays an important role in viral pathogenic processes. Numerous researches have reported that the sharp rise in ROS levels serve as one of the most significant changes in the microenvironment changes caused by viral infection. In our study, ROS have been shown to significantly promote PRV proliferation. PRV infection causes intracellular persistently high ROS levels by promoting mtCa^2+^-stimulated ROS generation and preventing Nrf2-medaited ROS clearance, which activates AMPK-ULK1 pathway mitophagy. Then, the activated mitophagy degrades the MAVS protein and inhibits the production of IFN I interferon, resulting in promoting self-replication (Figure 6).

Increased ROS levels inevitably affect the macromolecules and organelles within cells. They can oxidize and modify proteins, peroxidize lipids, damage nucleic acids, and even disrupt organelle homeostasis. Any changes to them are bound to have far-reaching effects. Viruses, as intracellular parasites, have evolved various strategies to utilize the microenvironment reshaped by ROS for their survival, such as altering substance metabolism, and interfering with the host immune response [16, 26]. During influenza A virus (IAV) [27] and respiratory syncytial virus infection (RSV) [28], elevated ROS levels activate HIF-1α-mediated glycolytic pathway to compensate for the energy deficit, contributing the replication. Porcine reproductive and respiratory syndrome virus (PRRSV) infection triggers ROS production to activate AKT/PCK1/INSIGs-mediated lipid synthesis for the enhancement of viral proliferation [29]. For Kaposi’s sarcoma-associated herpesvirus (KSHV), increased ROS levels contribute to spontaneous lytic replication or reactivation from latency and NAC treatment inhibits the above-mentioned processes [30]. Similarly, we also found that increased ROS levels were beneficial to the PRV replication. Furthermore, we found the facilitation of ROS on PRV resulted from promoting the degradation of MAVS and then impairing MAVS-mediated IFN-I responses (Figure 1). In our previous study, we confirmed that PRV infection induces mitophagy to degrade damaged mitochondria, and this process is accompanied by the degradation of MAVS protein [17]. Therefore, we speculated that ROS was an inducer of mitophagy during PRV infection. In the current study, after NAC treatment, PRV-induced mitochondria damage was alleviated and mitophagy was inhibited, which confirmed our speculation (Figure 2).

AMPK acts as a sensitive sensor of cell stress and is activated to affect mitochondrial homeostasis, autophagy, material metabolism (glucose or lipid metabolism) and other cellular processes during virus infection. AMPK has different activation states and plays different roles in different virus infection processes. During human cytomegalovirus (HCMV) and NDV infection, AMPK is activated to facilitate increased glycolytic flux, which creates favorable conditions for viral replication [31, 32]. Li et al. reported that RSV infection activates AMPK-mTOR-mediated autophagy through stimulating ROS production to favor the viral replication [33]. However, coxsackie virus B3 (CBV3) infection activates AMPK, resulting in restricted CVB3 replication through decreasing lipid amassing with the inhibition of lipid synthesis gene expression [34]. Interestingly, HSV-1 infection differentially regulates the phosphorylation of AMPK in neuronal cells [35]. At the early period of infection (< 4 hpi), HSV-1 inhibits AMPK/Sirt1 axis to activate p53-mediated apoptosis to achieve the anti-virus action, and after 4 hpi, the phosphorylation of AMPK is gradually increased to acetylate p53 and weaken apoptosis. Therefore, HSV-1 could manipulate AMPK to promote self-replication according to the needs of different infection periods. Therefore, HSV-1 is able to adapt to changes in AMPK and turn it into a promoter of self-replication. In our study, PRV infection notably increased the phosphorylation levels of both AMPK and ULK1, key regulators in the autophagy initiation cascade, while the phosphorylation of mTOR, a negative regulator of autophagy, remains unaffected. This phenomenon might be rationalized by considering the distinct signaling dynamics triggered by PRV. PRV infection likely activates the upstream signaling molecules that specifically target AMPK, leading to its phosphorylation at Thr172. Phosphorylated AMPK then directly phosphorylates ULK1, promoting the formation of the ULK1 complex, which is essential for the initiation of autophagy. Meanwhile, the lack of change in mTOR phosphorylation suggests that PRV might bypass or inhibit the upstream pathways that typically activate mTOR during cellular stress, ensuring that the autophagy process is not suppressed. This coordinated activation of the AMPK-ULK1 axis without mTOR interference allows for the efficient induction of autophagy. As our study demonstrated, increased ROS levels can increase the phosphorylation of AMPK at Thr172 both in total lysates and mitochondrial lysates, contributing to the activation of mitophagy, resulting in the facilitation of PRV infection (Figure 3).

Having clearly elucidated how ROS promote the replication of PRV, the mechanism by which PRV induces the production of ROS becomes the next question we aim to solve. In general, there are two main reasons for persistently high ROS levels in cells: excessive ROS production or delayed ROS clearance. Mitochondria are the main organelles of ROS production, and in turn, viruses, have evolved strategies to disrupt the redox balance maintained by mitochondria. Some viruses stimulate ROS production through interaction of viral proteins with mitochondrial proteins, inducing abnormal mtCa^2+^ flow and so on. The Vpr protein of HIV could inhibit mitochondrial fusion protein mitofusin 2 expressions causing MMP loss and ROS production [36]. The HBx protein of HBV has been reported to dysregulate mitochondrial permeability transition pore, which leads to MMP depolarization and ROS production [37]. Furthermore, it is also found that the endoplasmic Ca^2+^ abnormally flow into mitochondria, which is associated with ROS production [13]. During PRV infection, we found that increased mtCa^2+^ contributed to stimulate ROS generation and following the activation of AMPK (Figure 4). However, due to the large number of viral proteins in PRV, we do not currently find a viral protein that plays a dominant role in this process. In response to excessive ROS production, cells have inherent antioxidant systems to maintain the redox balance, like Nrf2 antioxidant system. In a mouse model, HSV-1 infection reduces Nrf2 protein expression by accelerating the ubiquitylation process, which leads to ferroptosis and viral encephalitis. The restoration of Nrf2 protein levels efficiently inhibits viral proliferation and alleviates viral encephalitis [38]. Schachtele et al. found that SFN treatment can effectively mitigate HSV-1-induced neurotoxicity through decreasing ROS levels [39]. In our study, we found that PRV infection also inhibited Nrf2-mediated antioxidant system by blocking the nuclear translocation of Nrf2 instead of accelerating the degradation. Then, SFN was utilized to enhance the nuclear translocation during PRV infection, which exhibited an inhibitory effect on the viral replication (Figure 5).

Our research has one limitation in that it lacks an in-depth animal model study. Future research should prioritize the establishment of mice/pig models to validate the role of oxidative stress in the PRV pathogenic processes and provides a theoretical basis for seeking ROS-based antiviral therapeutic strategy.

In summary, our study uncovers the mechanism of ROS generation during PRV infection and identifies elevated ROS levels as a critical upstream event that promotes PRV-induced mitophagic degradation of MAVS, thereby facilitating viral replication. This work highlights the role of oxidative stress in PRV pathogenesis and proposes a potential therapeutic strategy for controlling PRV infection.

## Data Availability

All data generated or analyzed during this study are contained within the manuscript.

## Abbreviations

ROS: reactive oxygen species
PRV: pseudorabies virus
ER: endoplasmic reticulum
mtCa^2+^: mitochondrial Ca^2+^
MMP: mitochondrial membrane potential
MAVS: mitochondrial antiviral signaling protein
NAC: N-acetyl-L-cysteine
IFIT1: IFN-stimulated genes tetratricopeptide repeats 1
AMPK: adenosine5’-monophosphate (AMP)-activated protein kinase
MCU: mitochondrial calcium uniporter

## References

[1] Ha HL, Shin HJ, Feitelson MA, Yu DY (2010) Oxidative stress and antioxidants in hepatic pathogenesis. World J Gastroenterol 16:6035–6043 21182217 10.3748/wjg.v16.i48.6035 PMC3012582

[2] Nencioni L, Sgarbanti R, De Chiara G, Garaci E, Palamara AT (2007) Influenza virus and redox mediated cell signaling: a complex network of virus/host interaction. New Microbiol 30:367–375 18080671

[3] Protto V, Tramutola A, Fabiani M, Marcocci ME, Napoletani G, Iavarone F, Vincenzoni F, Castagnola M, Perluigi M, Di Domenico F, De Chiara G, Palamara AT (2020) Multiple herpes simplex virus-1 (HSV-1) reactivations induce protein oxidative damage in mouse brain: novel mechanisms for Alzheimer’s disease progression. Microorganisms 8:972 32610629 10.3390/microorganisms8070972 PMC7409037

[4] Hu J, Li H, Luo X, Li Y, Bode A, Cao Y (2017) The role of oxidative stress in EBV lytic reactivation, radioresistance and the potential preventive and therapeutic implications. Int J Cancer 141:1722–1729 28571118 10.1002/ijc.30816

[5] Sun W, Liu S, Yan Y, Wang Q, Fan Y, Okyere SK (2023) Pseudorabies virus causes splenic injury via inducing oxidative stress and apoptosis related factors in mice. Sci Rep 13:23011 38155259 10.1038/s41598-023-50431-7 PMC10754911

[6] Lai IH, Chang CD, Shih WL (2019) Apoptosis induction by pseudorabies virus via oxidative stress and subsequent DNA damage signaling. Intervirology 62:116–123 31430757 10.1159/000502047

[7] Huan C, Xu Y, Zhang W, Pan H, Zhou Z, Yao J, Guo T, Ni B, Gao S (2022) Hippophae rhamnoides polysaccharides dampen pseudorabies virus infection through downregulating adsorption, entry and oxidative stress. Int J Biol Macromol 207:454–463 35278510 10.1016/j.ijbiomac.2022.03.041

[8] Wang Q, Xie X, Chen Q, Yi S, Chen J, Xiao Q, Yu M, Wei Y, Hu T (2022) Effects of quercitrin on PRV-induced secretion of reactive oxygen species and prediction of lncRNA regulatory targets in 3D4/2 cells. Antioxidants 11:631 35453316 10.3390/antiox11040631 PMC9031018

[9] Lennicke C, Cochemé HM (2021) Redox metabolism: ROS as specific molecular regulators of cell signaling and function. Mol Cell 81:3691–3707 34547234 10.1016/j.molcel.2021.08.018

[10] Liu S, Pi J, Zhang Q (2022) Signal amplification in the KEAP1-NRF2-ARE antioxidant response pathway. Redox Biol 54:102389 35792437 10.1016/j.redox.2022.102389 PMC9287733

[11] Piccoli C, Scrima R, Quarato G, D’Aprile A, Ripoli M, Lecce L, Boffoli D, Moradpour D, Capitanio N (2007) Hepatitis C virus protein expression causes calcium-mediated mitochondrial bioenergetic dysfunction and nitro-oxidative stress. Hepatology 46:58–65 17567832 10.1002/hep.21679

[12] Lecoeur H, Borgne-Sanchez A, Chaloin O, El-Khoury R, Brabant M, Langonné A, Porceddu M, Brière JJ, Buron N, Rebouillat D, Péchoux C, Deniaud A, Brenner C, Briand JP, Muller S, Rustin P, Jacotot E (2012) HIV-1 Tat protein directly induces mitochondrial membrane permeabilization and inactivates cytochrome c oxidase. Cell Death Dis 3:e282 22419111 10.1038/cddis.2012.21 PMC3317353

[13] Choi YM, Lee SY, Kim BJ (2019) Naturally occurring hepatitis B virus mutations leading to endoplasmic reticulum stress and their contribution to the progression of hepatocellular carcinoma. Int J Mol Sci 20:597 30704071 10.3390/ijms20030597 PMC6387469

[14] Fu PF, Cheng X, Su BQ, Duan LF, Wang CR, Niu XR, Wang J, Yang GY, Chu BB (2021) CRISPR/Cas9-based generation of a recombinant double-reporter pseudorabies virus and its characterization in vitro and in vivo. Vet Res 52:95 34174954 10.1186/s13567-021-00964-4 PMC8233574

[15] Lian Z, Liu P, Zhu Z, Sun Z, Yu X, Deng J, Li R, Li X, Tian K (2023) Isolation and characterization of a novel recombinant classical pseudorabies virus in the context of the variant strains pandemic in China. Viruses 15:1966 37766372 10.3390/v15091966 PMC10536572

[16] Foo J, Bellot G, Pervaiz S, Alonso S (2022) Mitochondria-mediated oxidative stress during viral infection. Trends Microbiol 30:679–692 35063304 10.1016/j.tim.2021.12.011

[17] Zhao Y, Ding C, Zhu Z, Wang W, Wen W, Favoreel HW, Li X (2024) Pseudorabies virus infection triggers mitophagy to dampen the interferon response and promote viral replication. J Virol 98:e0104824 39212384 10.1128/jvi.01048-24 PMC11494983

[18] Hinchy EC, Gruszczyk AV, Willows R, Navaratnam N, Hall AR, Bates G, Bright TP, Krieg T, Carling D, Murphy MP (2018) Mitochondria-derived ROS activate AMP-activated protein kinase (AMPK) indirectly. J Biol Chem 293:17208–17217 30232152 10.1074/jbc.RA118.002579 PMC6222118

[19] Iorio R, Celenza G, Petricca S (2021) Mitophagy: molecular mechanisms, new concepts on Parkin activation and the emerging role of AMPK/ULK1 Axis. Cells 11:30 35011593 10.3390/cells11010030 PMC8750607

[20] Hung CM, Lombardo PS, Malik N, Brun SN, Hellberg K, Van Nostrand JL, Garcia D, Baumgart J, Diffenderfer K, Asara JM, Shaw RJ (2021) AMPK/ULK1-mediated phosphorylation of Parkin ACT domain mediates an early step in mitophagy. Sci Adv 7:4544 10.1126/sciadv.abg4544 PMC8026119 33827825

[21] Drake JC, Wilson RJ, Laker RC, Guan Y, Spaulding HR, Nichenko AS, Shen W, Shang H, Dorn MV, Huang K, Zhang M, Bandara AB, Brisendine MH, Kashatus JA, Sharma PR, Young A, Gautam J, Cao R, Wallrabe H, Chang PA, Wong M, Desjardins EM, Hawley SA, Christ GJ, Kashatus DF, Miller CL, Wolf MJ, Periasamy A, Steinberg GR, Hardie DG, Yan Z (2021) Mitochondria-localized AMPK responds to local energetics and contributes to exercise and energetic stress-induced mitophagy. Proc Natl Acad Sci USA 118:e2025932118 34493662 10.1073/pnas.2025932118 PMC8449344

[22] Zhao H, Pan X (2021) Mitochondrial Ca(2+) and cell cycle regulation. Int Rev Cell Mol Biol 362:171–207 34253295 10.1016/bs.ircmb.2021.02.015

[23] Lee C (2018) Therapeutic modulation of virus-induced oxidative stress via the Nrf2-dependent antioxidative pathway. Oxid Med Cell Longev 2018:6208067 30515256 10.1155/2018/6208067 PMC6234444

[24] He F, Ru X, Wen T (2020) NRF2, a transcription factor for stress response and beyond. Int J Mol Sci 21:4777 32640524 10.3390/ijms21134777 PMC7369905

[25] Espinosa-Diez C, Miguel V, Mennerich D, Kietzmann T, Sánchez-Pérez P, Cadenas S, Lamas S (2015) Antioxidant responses and cellular adjustments to oxidative stress. Redox Biol 6:183–197 26233704 10.1016/j.redox.2015.07.008 PMC4534574

[26] Gruhne B, Sompallae R, Marescotti D, Kamranvar SA, Gastaldello S, Masucci MG (2009) The Epstein-Barr virus nuclear antigen-1 promotes genomic instability via induction of reactive oxygen species. Proc Natl Acad Sci USA 106:2313–2318 19139406 10.1073/pnas.0810619106 PMC2650153

[27] Zhang Y, Chang L, Xin X, Qiao Y, Qiao W, Ping J, Xia J, Su J (2024) Influenza A virus-induced glycolysis facilitates virus replication by activating ROS/HIF-1α pathway. Free Radic Biol Med 225:910–924 39491735 10.1016/j.freeradbiomed.2024.10.304

[28] Chen LF, Cai JX, Zhang JJ, Tang YJ, Chen JY, Xiong S, Li YL, Zhang H, Liu Z, Li MM (2023) Respiratory syncytial virus co-opts hypoxia-inducible factor-1α-mediated glycolysis to favor the production of infectious virus. MBio 14:e0211023 37796013 10.1128/mbio.02110-23 PMC10653832

[29] Ma YX, Han YQ, Wang PZ, Wang MY, Yang GY, Li JL, Wang J, Chu BB (2024) Porcine reproductive and respiratory syndrome virus activates lipid synthesis through a ROS-dependent AKT/PCK1/INSIG/SREBPs axis. Int J Biol Macromol 282:136720 39433189 10.1016/j.ijbiomac.2024.136720

[30] Ye F, Zhou F, Bedolla RG, Jones T, Lei X, Kang T, Guadalupe M, Gao SJ (2011) Reactive oxygen species hydrogen peroxide mediates Kaposi’s sarcoma-associated herpesvirus reactivation from latency. PLoS Pathog 7:e1002054 21625536 10.1371/journal.ppat.1002054 PMC3098240

[31] McArdle J, Moorman NJ, Munger J (2012) HCMV targets the metabolic stress response through activation of AMPK whose activity is important for viral replication. PLoS Pathog 8:e1002502 22291597 10.1371/journal.ppat.1002502 PMC3266935

[32] Gong Y, Tang N, Liu P, Sun Y, Lu S, Liu W, Tan L, Song C, Qiu X, Liao Y, Yu S, Liu X, Lin SH, Ding C (2022) Newcastle disease virus degrades SIRT3 via PINK1-PRKN-dependent mitophagy to reprogram energy metabolism in infected cells. Autophagy 18:1503–1521 34720029 10.1080/15548627.2021.1990515 PMC9298456

[33] Li M, Li J, Zeng R, Yang J, Liu J, Zhang Z, Song X, Yao Z, Ma C, Li W, Wang K, Wei L (2018) Respiratory syncytial virus replication is promoted by autophagy-mediated inhibition of apoptosis. J Virol 92:e02193-e2217 29386287 10.1128/JVI.02193-17 PMC5874425

[34] Xie W, Wang L, Dai Q, Yu H, He X, Xiong J, Sheng H, Zhang D, Xin R, Qi Y, Hu F, Guo S, Zhang K (2015) Activation of AMPK restricts coxsackievirus B3 replication by inhibiting lipid accumulation. J Mol Cell Cardiol 85:155–167 26055448 10.1016/j.yjmcc.2015.05.021

[35] Martin C, Leyton L, Arancibia Y, Cuevas A, Zambrano A, Concha MI, Otth C (2014) Modulation of the AMPK/Sirt1 axis during neuronal infection by herpes simplex virus type 1. J Alzheimers Dis 42:301–312 24858404 10.3233/JAD-140237

[36] Huang CY, Chiang SF, Lin TY, Chiou SH, Chow KC (2012) HIV-1 Vpr triggers mitochondrial destruction by impairing Mfn2-mediated ER-mitochondria interaction. PLoS One 7:e33657 22438978 10.1371/journal.pone.0033657 PMC3306277

[37] Rahmani Z, Huh KW, Lasher R, Siddiqui A (2000) Hepatitis B virus X protein colocalizes to mitochondria with a human voltage-dependent anion channel, HVDAC3, and alters its transmembrane potential. J Virol 74:2840–2846 10684300 10.1128/jvi.74.6.2840-2846.2000 PMC111774

[38] Xu XQ, Xu T, Ji W, Wang C, Ren Y, Xiong X, Zhou X, Lin SH, Xu Y, Qiu Y (2023) Herpes simplex virus 1-induced ferroptosis contributes to viral encephalitis. mBio 14:e0237022 36507835 10.1128/mbio.02370-22 PMC9973258

[39] Schachtele SJ, Hu S, Lokensgard JR (2012) Modulation of experimental herpes encephalitis-associated neurotoxicity through sulforaphane treatment. PLoS One 7:e36216 22558388 10.1371/journal.pone.0036216 PMC3338688

